# DyNeuMo Mk-2: An Investigational Circadian-Locked Neuromodulator with Responsive Stimulation for Applied Chronobiology

**DOI:** 10.1109/SMC42975.2020.9283187

**Published:** 2020-12-14

**Authors:** Robert Toth, Mayela Zamora, Jon Ottaway, Tom Gillbe, Sean Martin, Moaad Benjaber, Guy Lamb, Tara Noone, Barry Taylor, Alceste Deli, Vaclav Kremen, Gregory Worrell, Timothy G. Constandinou, Ivor Gillbe, Stefan De Wachter, Charles Knowles, Andrew Sharott, Antonio Valentin, Alexander L. Green, Timothy Denison

**Affiliations:** MRC Brain Network Dynamics Unit, and the Department of Engineering Science, University of Oxford, Oxford OX2 7DQ, UK; MRC Brain Network Dynamics Unit, and the Department of Engineering Science, University of Oxford, Oxford OX2 7DQ, UK; Bioinduction Ltd, Bristol BS8 4RP, UK; Bioinduction Ltd, Bristol BS8 4RP, UK; Department of Neurosurgery, John Radcliffe Hospital, Oxford OX3 9DU, UK; MRC Brain Network Dynamics Unit, and the Department of Engineering Science, University of Oxford, Oxford OX2 7DQ, UK; Bioinduction Ltd, Bristol BS8 4RP, UK; Bioinduction Ltd, Bristol BS8 4RP, UK; Bioinduction Ltd, Bristol BS8 4RP, UK; Department of Neurosurgery, John Radcliffe Hospital, Oxford OX3 9DU, UK; Bioelectronics Neurophysiology and Engineering Lab, Mayo Clinic, Rochester, MN, US; Bioelectronics Neurophysiology and Engineering Lab, Mayo Clinic, Rochester, MN, US; Department of Electrical and Electronic Engineering and the UK Dementia Research Institute (Care Research and Technology Centre), Imperial College London, London SW7 2AZ, UK; Bioinduction Ltd, Bristol BS8 4RP, UK; Department of Urology, University of Antwerp Hospital, 2650 Edegem, Belgium; Barts and the London School of Medicine and Dentistry, Queen Mary University of London, London E1 2AT, UK; MRC Brain Network Dynamics Unit, and the Department of Engineering Science, University of Oxford, Oxford OX2 7DQ, UK; Department of Basic and Clinical Neuroscience, King’s College London, London SE5 9RT, UK; Department of Neurosurgery, John Radcliffe Hospital, Oxford OX3 9DU, UK; MRC Brain Network Dynamics Unit, and the Department of Engineering Science, University of Oxford, Oxford OX2 7DQ, UK

**Keywords:** Neural implants, Brain stimulation, Activity recognition, Digital filters, Adaptive control, Closed loop systems, Chronobiology, Circadian rhythm, Safety management

## Abstract

Deep brain stimulation (DBS) for Parkinson’s disease, essential tremor and epilepsy is an established palliative treatment. DBS uses electrical neuromodulation to suppress symptoms. Most current systems provide a continuous pattern of fixed stimulation, with clinical follow-ups to refine settings constrained to normal office hours. An issue with this management strategy is that the impact of stimulation on circadian, i.e. sleep-wake, rhythms is not fully considered; either in the device design or in the clinical follow-up. Since devices can be implanted in brain targets that couple into the reticular activating network, impact on wakefulness and sleep can be significant. This issue will likely grow as new targets are explored, with the potential to create entraining signals that are uncoupled from environmental influences. To address this issue, we have designed a new brain-machine-interface for DBS that combines a slow-adaptive circadian-based stimulation pattern with a fast-acting pathway for responsive stimulation, demonstrated here for seizure management. In preparation for first-in-human research trials to explore the utility of multi-timescale automated adaptive algorithms, design and prototyping was carried out in line with ISO risk management standards, ensuring patient safety. The ultimate aim is to account for chronobiology within the algorithms embedded in brain-machine-interfaces and in neuromodulation technology more broadly.

## I Introduction

Neuromodulation is effective for treating several disorders of the nervous system including movement disorders and epilepsy. Current neuromodulation systems tend to apply tonic, fixed patterns that change only with user intervention through an external patient controller. While generally effective, these systems do not actively respond to changes in patient state, such as pharmacological perturbations, sleep or activity. One exception is responsive neurostimulation (RNS) for epilepsy by Neuropace, which responds to electrical biomarkers in the brain and stimulates in response to pre-configured classification states [1]. The ability to sense biomarkers in RNS has shown that neurological symptoms might follow periodic rhythms. More specifically, an analysis of RNS data logs revealed that circadian and infradian (multi-day) patterns in seizure behavior might be relevant for optimizing treatment [2]. These rhythms have been verified in humans and canines with epilepsy [3], [4]. In addition, patients with vagal nerve stimulators can show an increased incidence of sleep apnea [5]; to address this side-effect, devices such as the SenTiva from LivaNova can adjust stimulation parameters based on the time of day.

Emerging data suggests that circadian rhythms, and chronobiology in general, might prove important in the treatment of disease states. Chronobiology is defined in this paper as time-based cycles in physiology, arising from evolutionarily preserved internal temporal rhythms, along with their modulation upon anticipation of environmental change. Pharmaceutical control methods are actively being explored to adjust and restore circadian cycles following external or pathological disruptions [6]. Stimulation-based interventions targeting neural regulatory centers of these cycles can also directly impact behavior. For instance, stimulation of brain structures coupled with the reticular activating network can impact sleep architecture and wakefulness. One example of this issue is stimulation of the anterior nucleus for epilepsy [7]. Likewise, stimulation of the pedunculopontine nucleus (PPN) for gait in Parkinson’s disease and multiple system atrophy appears to impact sleep/wake patterns [8], [9]. Other brain targets being explored for therapy, such as the hypothalamus for chronic pain and cluster headaches [10], and the centromedian thalamus (CMT) for Lennox-Gastaut epilepsy [11], are also expected to modulate sleep/wake behavior [12]. In summary, the brain circuits stimulated for therapy often couple into the reticular activating network; arguably, neuromodulation’s impact on circadian rhythms is not fully considered in brain-machine-interface and therapy design.

We propose modulating brain networks synchronous with circadian rhythms. As opposed to fixed “tonic” stimulation, we wish to apply a circadian pattern to modulate brain behavior in a more neurotypical state. However, emergent symptoms such as a change of posture/activity (e.g. getting up for the bathroom at night, or a breakthrough seizure) also warrant “fast-acting” stimulation to modify the slowly adapting stimulation rhythm, much like exposure to environmental triggers can phase-reset circadian patterns. To explore this concept, we designed a human-implantable research tool that allows for this dual-mode algorithmic control.

**Fig. 1:**
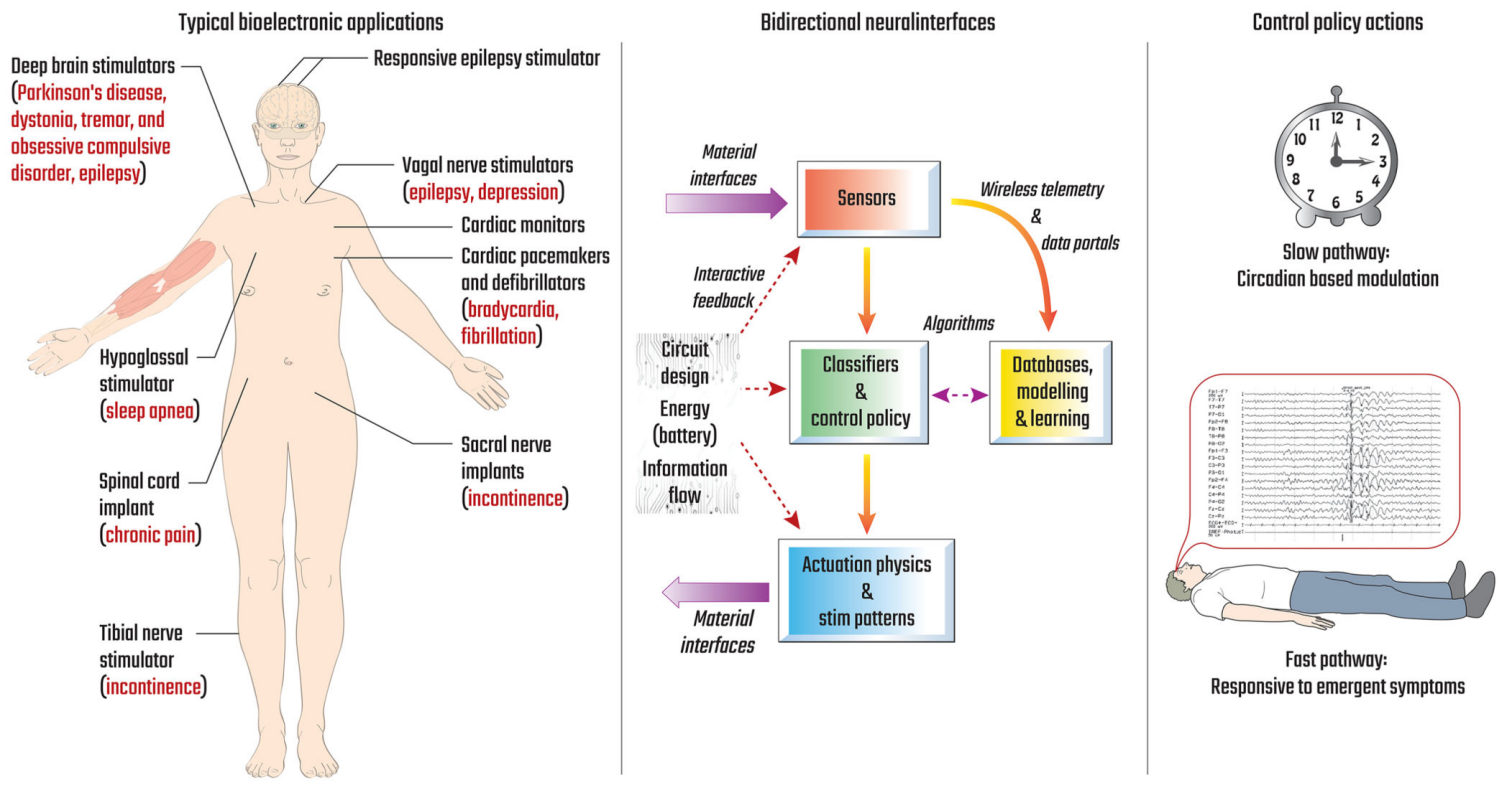
Overview of the DyNeuMo Mk-2 program. Left: Target applications considered for the user requirements and risk management. Middle: Technology stack sub-components required for system integration. Right: Dual-mode control policies, which are the focus of this research tool. Adapted from [13].

In this paper we build on the work of the Dynamic Neuro-Modulator (DyNeuMo) Mk-1, presented in [14]. The resulting DyNeuMo Mk-2, illustrated in Fig. 1, superimposes slowadaptive (applied chronobiology) and fast-adaptive (responsive to physiomarker) algorithms to adapt stimulation settings. To support first-in-human research for several applications, we used ISO 13485-compliant design controls throughout the project. Supporting this structure, the paper starts with the assessment of our device requirements anticipating user needs and risk management. We then discuss the implementation of our design before demonstrating the system’s functionality through verification testing. We conclude with a description of upcoming research projects where the mixed-mode control might prove particularly effective, as well as a discussion of the limitations of the research tool. In summary, the DyNeuMo Mk-2 research stimulator aims to further extend chronobiology research into neuromodulation, providing patient-specific therapies based on intrinsic time-based rhythms, in addition to immediate symptoms and activities.

## II System Requirements Overview

The DyNeuMo Mk-2 system was designed to be a flexible research tool for designing automated interventions that merge slow- and fast-adaptive algorithms. The research tool requirements emphasizing the algorithms are summarized in Table I, building on the system-level requirements discussed in [14].

When creating the DyNeuMo platform, a critical design requirement was that reserach features could not compromise capabilities offered by clinically approved therapy systems, in line with other state-of-the-art research projets [15], [16]. The platform is designed to match the capabilities of common deep brain, chronic pain, sacral (incontinence), and gastric stimulators based on publicly-available manufacturer specifications, as stated in [14]. The focus of this paper is on brain therapies.

For the slow-adaptive algorithm, we aim to provide a 24-hour timing cycle with acceptable granularity to adjust stimulation based on the time of day. This requirement brings trade-offs of user configuration burden versus specificity – a 30 minute resolution was chosen. The user must also be able to map predefined stimulation parameters to these time epochs in a simple manner. We also support two complementary modes of fast-adaptive algorithms. The first, introduced in the Mk-1 system, aims to capture motion-based states such as general activity, gait and freezing, posture and falls. The clinicinan-researcher can set up the detection of these inertial states to explore and improve therapies for movement disorders [18], or to account for postural effects in orthostatic hypertension [14]. As well as automatic stimulation adaptation, inertial sensing can be used both for delivering automatic stimulation adjustments, and to provide important diagnostic information on the patient [14]. The other basis for fast-adaptation, novel in the Mk-2 system, is bioelectric activity measured directly from the implanted electrodes – this can help detect events such as electrographic seizures in epilepsy [1] or the akinetic or dyskinetic states in movement disorders [19], [20].

The adaptive nature of the system introduces design requirements of its own. Importantly, individualized data collection is required to allow for patient-specific training of state-classifiers. In addition, the algorithm implementation should not materially affect power consumption compared to baseline therapy stimulation. As a benchmark, the device typically consumes approximately 400μW for typical bilateral deep brain stimulation therapy [14]. However, we also aim to facilitate research, which can require increased power for data collection and algorithm prototyping. To support this, we specified a rechargeable system. Our lead users conveyed that they are willing to accept a daily recharge to explore advanced algorithms that might significantly improve therapy outcomes. Finally, we require a safe verification process to confirm the functional operation of the adaptive algorithm in each patient prior to release from the controlled clinical setting.

## III System Design

**Fig. 2:**
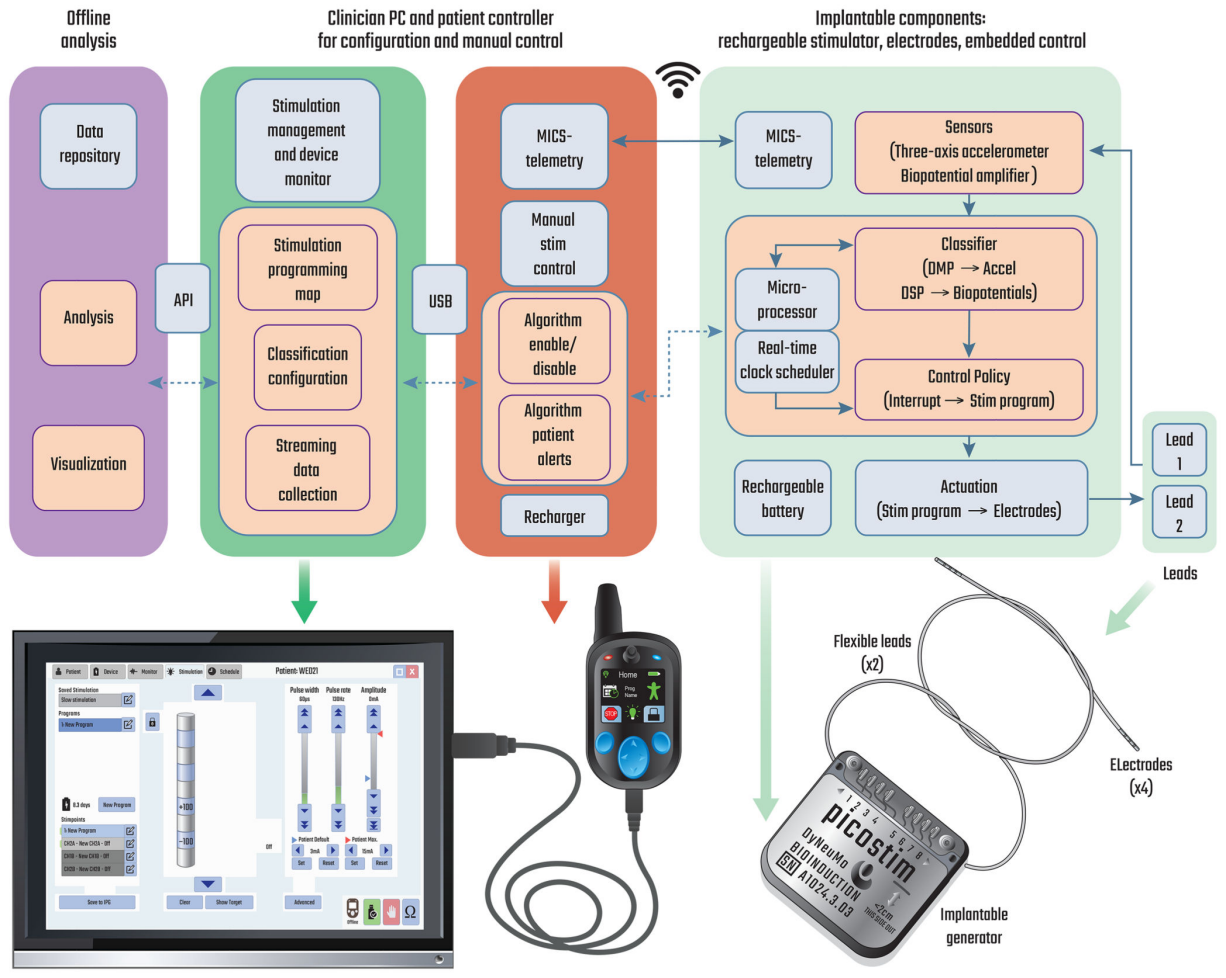
Top: system block diagram for the DyNeuMo Mk-2 supplementing the baseline functionality provided by the predicate Picostim with the addition of the slow- and fast-adapting algorithms. (Acronyms: API is an application programming interface, MICS is the Medical Information and Communication band) Bottom: actual physical components for the DyNeuMo research system. Note that the research tool is upgradeable through the firmware and software versions, while mechanical components are largely reused. The USB connector between the patient programmer and tablet is for in-clinic programming. Research subjects use the handheld controller for at-home recharge and manual adjustments. Adapted from [14].

The DyNeuMo platform was designed using a physiological control model [14], [17]. The system block diagram shown in Fig. 2 outlines the control pathway and user interfaces; a few areas of detail are provided here for context. Overall, our aim is to automatically titrate stimulation parameters using a combination of slow-adapting, timer-scheduled adjustments supplemented with fast-adaptive changes based on motion or bioelectrical classifications. These can be considered as complementary, parallel response loops. Using this framework, we integrated the full technology stack for a bi-directional neural interface with multiple sensors, classifiers, and stimulation actuation; the control policy uses schedule-based parameter control supplemented with fast updates from the physiological sensors. The adjunct patient controller assists with emergency algorithm intervention and recharge, while the clinician programmer is used for algorithm training and configuration.

The project followed the ISO 14971 risk management process to ensure patient safety. The IEC 60601-1-10 framework was taken as reference for the algorithm risk mitigations [17]. While the considerations are detailed in [14], a brief summary is presented here. To mitigate the risks of automatic parameter changes, actuation-limits are constrained to known-safe levels, as defined by the programming clinician. Stimulation-state transitions are ramped in order to avoid paresthesia [21]. Further, the patient can, if needed, disable the adaptive algorithm with a button press on their controller, and enter a predefined open-loop “fallback” mode [14].

Working through the technology stack, the core stimulator functionality is provided by circuits from the Picostim from Bioinduction, the predicate device on which our research system is based [22]. Two independent current sources can drive patterns of stimulation over a range from 1 Hz to 450 Hz. Cycling of stimulation allows for bursts of activity and sub-Hz functionality. The current output levels and other parameters are summarized in [14], and align with common neuromodulation specifications.

For sensing and classification, we consider three algorithmic pathways. The slow-adaptive system uses a 24-hour scheduler that maps stimulation parameters to time-based epochs. The scheduler uses the real-time clock of the embedded microprocessor to signal when a change is due, and sets the pointer to the new stimulation parameters to be loaded into the stimulation parameter block. Up to eight programs are available to the scheduler, but in practice the user will likely reserve two unique patterns for the fast-adaptive algorithms.

The fast adapting algorithms use either motion or bioelectrical sensing inputs; at this time, the user must select between the two as a safety measure. The motion-adaptive pathway was introduced in DyNeuMo Mk-1 [14], a summary of features follows. Inertial sensing is provided by an embedded ADXL346 microelectromechanical accelerometer, with an integrated digital motion processor (DMP) for motion state classification [23]. Sensing and classifier options are fully configurable through the in-clinic tablet programmer. To lower programming burden on the clinician, reference parameter sets are provided for common use cases [14].

The bioelectrical sensing pathway introduced in the DyNeuMo Mk-2 uses a chopper-stabilized instrumentation amplifier to detect potentials off an electrode dipole. Chopper-stabilization provides greater resolution to low-frequency oscillations in the brain networks [15], [16]. The dipole can be configured with a multiplexer to select any combination of electrodes, including the device case. The signal sampling is synchronous with the stimulation pattern, including a brief blanking interval during the stimulation pulse, in which the sensing signal state is preserved [24]. This strategy helps maintain the resolution floor of the sensing chain in the presence of stimulation. The sensing channel is digitized at a multiple of the stimulation frequency, with a minimum of 5× greater sampling rate, depending on the biomarkers of interest. The data stream is then processed with a digital signal processing algorithm that can be configured from the clinician interface. The flexible algorithm approach allows classification of the signal using methods similar to those published for closed-loop devices for Parkinson’s disease [19], [25], [26] and epilepsy control systems [1].

As a control policy, the classifier algorithms may raise interrupts in the stimulation engine, triggering a transition between preset programs. An important constraint is mandating ramped transitions between stimulation patterns, in order to reduce side-effects such as paresthesia [21]. The ramp rate, the stimulation program associated to each trigger, as well as a default “fallback” program for risk management are all configured by the clinician.

Given the presence of multiple algorithms and adaptive pathways, their priorities had to be established. As a baseline, the stimulation program can be selected by allowing the latest interrupt to overwrite the current state. While this would satisfy our initial use cases, an intuitive hierarchy of interrupts is introduced to make operation unambiguous. Fast-adaptive changes must be given priority over the slow-adaptive circadian baseline to allow responsive operation. The highest precedence is then given to manual intervention, to allow the patient to change and even disable stimulation at will, as an important safety consideration.

The patient programmer provided to clinicians allows for extensive verification. Wireless telemetry is provided through MICS-band radio between the programmer and the implant, supporting real-time streaming of both accelerometer and biopotential data. State changes initiated by either the patient or the adaptive algorithms are telemetered and visible on the interface, allowing the clinician to easily verify settings. Additionally when telemetry is not active, the implantable is capable of recording field potential signals from the implanted electrodes as well as accelerometer data. This measurement mode offers an improved noise floor for establishing high quality baseline characteristics, and could also serve to validate the expected out-of-clinic performance of the system.

## IV System Verification

Thorough system verification is essential to ensure automated stimulation adjustments are delivered safely. Since much of the system hardware and software leverages the Picostim predicate [22], we could reuse the verification tests for existing attributes such as stimulation, telemetry, and bio-compatibility [14]. The incremental verification efforts focused on sensing and algorithms, specifically the performance of the fast-adaptive algorithms. Three representative use-cases are illustrated in this section: slow-adapting circadian patterns, motion-adapted stimulation, and bioelectric-based seizure-responsive stimulation. Results are summarized in Table II.

**Fig. 3:**
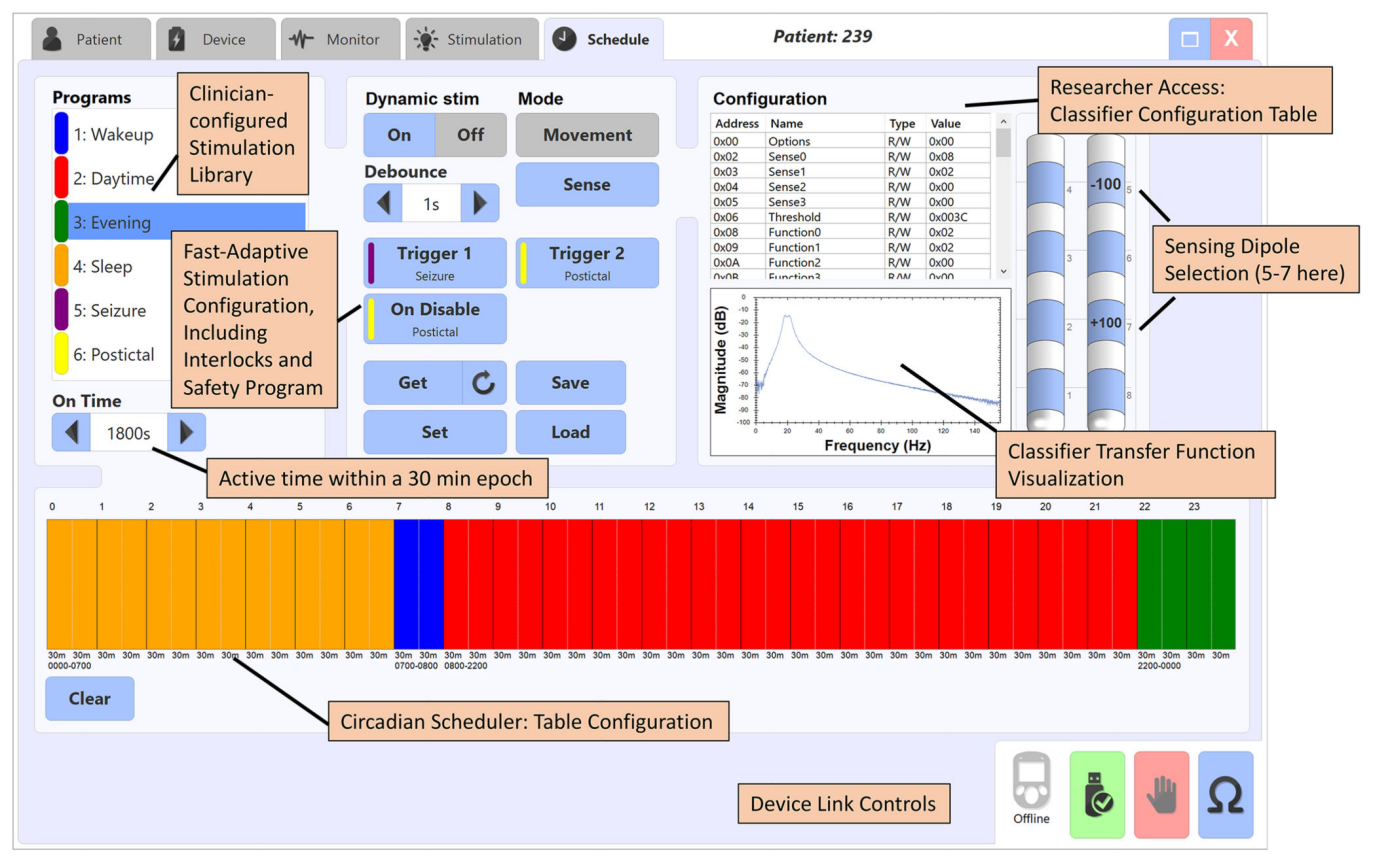
Screen shot of the algorithm configuration tab illustrating how the circadian table is configured for a 24-hour cycle. Note that the fast-adaptive algorithms are also included in the same module. Stimulation programs are mapped to classified states and time-based epochs.

The schedule-based algorithm was verified by assigning distinct patterns into the clinician programmer and running the device through a twenty-four hour cycle. Fig. 3 illustrates the clinician programmer interface and how the pattern is assigned. Note that the algorithm interface tab also includes the motion and bioelectrical adaptive configuration settings, as annotated in the figure.

Our previous work has demonstrated that the DMP can be programmed appropriately for detection of certain activity states. For the DyNeuMo Mk-1 we verified tap/shock detection, which could be useful for detecting transient events such as steps or falls, or as a mechanical patient input that eliminates their need for interaction with the handheld controller [14].

**Fig. 5:**
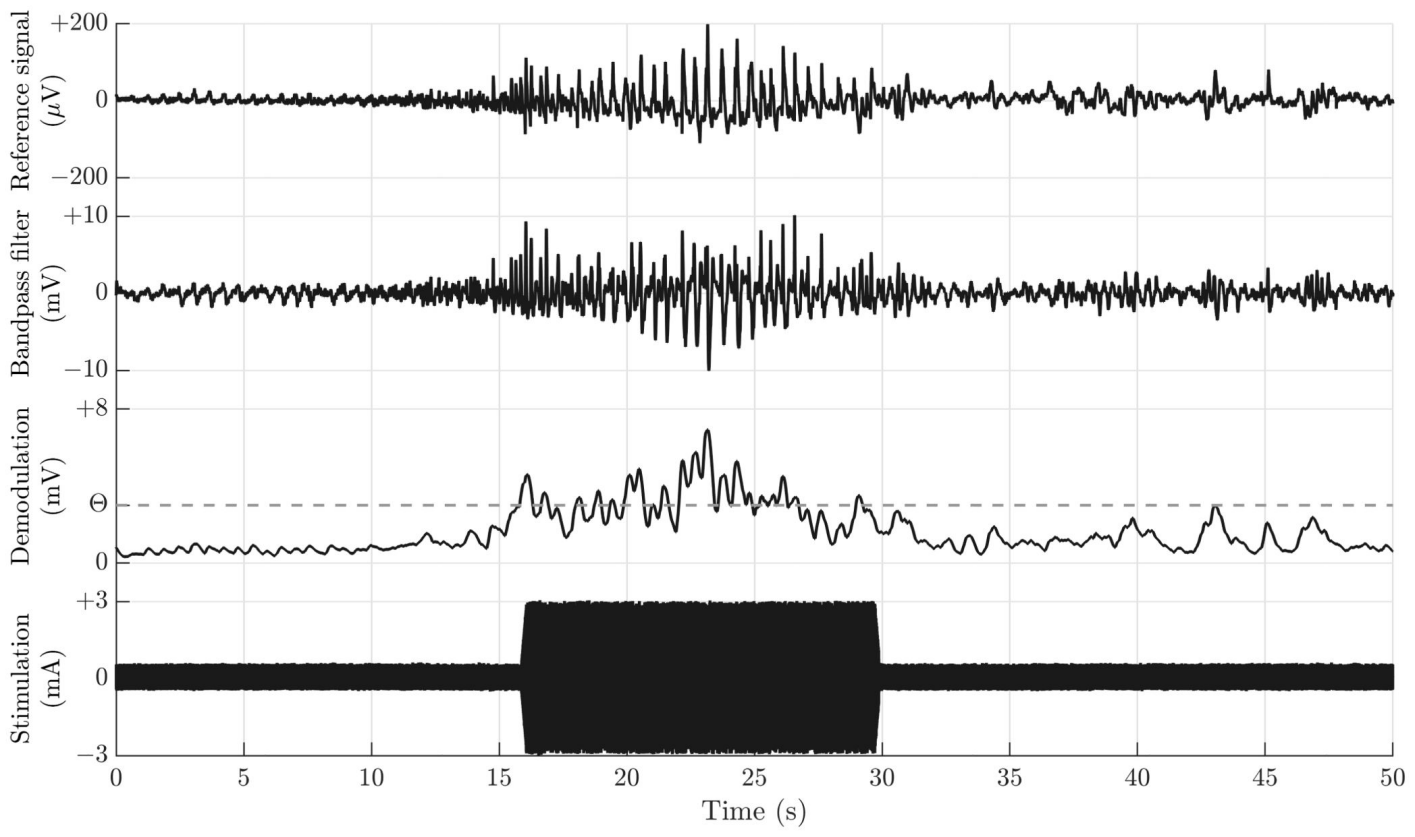
Demonstration of stimulation modulation based on real-time seizure detection. Top: input signal from pre-recorded human seizure from the anterior nucleus of the thalamus. Middle panels: Tuned band-pass filter (1–20 Hz, 4th order), and rectified/low-passed output to extract the envelope. Bottom: stimulation increases (from 0.5 to 3 mA) in response to a detected seizure for a pre-set period of 15 s in this example, upon crossing a clinician-adjustable threshold (Θ).

Bioelectrical-based verification protocols demonstrated that the electrophysiological sensing pathway could measure field potentials in the presence of stimulation and take appropriate actions. The system was integrated into a test bench that recreates the tissue-electrode interface, and pre-recorded signals from the anterior nucleus of a human thalamus provided to mimic physiologic activity; the signal levels were titrated to match the original data. Fig. 5 illustrates a typical use case for the system, where an emergent seizure is detected using algorithm methods consistent with previous studies [27], [28].

With our synchronized sense-stimulation electronics, bio-electrical signal classification is still feasible in the presence of stimulation, allowing for detection of seizure onset and transient adaptation of stimulation throughout the ictal period, and then cessation of this state-based stimulation at the termination of the seizure. The resolution for recording field potentials was characterized as slightly below 1 μVrms in the presence of stimulation, which is acceptable for challenging sub-cortical brain interfaces in the basal ganglia and thalamus, and provides significant margin for cortical signals and seizure detection. Note that this bench experiment does not capture the brain’s physiological response to stimulation, and is therefore limited to verifying the bi-directional interface’s technical characteristics. For future extensibility, the verified signal levels and algorithm characteristics are similar to those for cough and pelvic floor EMG, suggesting utility for peripheral applications in the future [29].

The power consumption of the accelerometer and motion classification is approximately 40μW, or 10% of the nominal therapeutic settings for a Parkinson’s disease or Essential tremor patient, as reported in [14]. The bioelectrical signal processing circuit is designed for flexibility and capability at this time, and not yet optimized for power; it runs for approximately thirty hours on a typical battery charge to support a daily cycle with buffer. Note that these estimates do not include potential energy savings from adaptive stimulation.

## V Next: Validation Through Clinical Trials

Ultimately, the impact of the algorithmic strategy must be evaluated on specific neurological conditions. To facilitate such clinical testing, as with the DyNeuMo Mk-1, the Mk-2 system is being released as a research tool for the clinical neuroscience community. Support similarly includes the design history files required for investigational device approvals. To mitigate the risks of using the DyNeuMo Mk-2 device, the research capabilities can once again be easily disabled, allowing the patient to benefit at a minimum from predicate therapy capabilities [14].

As a representative application, we are exploring the treatment of Lennox-Gastaut epilepsy through stimulation of the centromedian thalamus [11]. While promising results for stimulation have been observed in pilot studies [30], the circadian rhythm of seizure rates, and how to optimize stimulation for seizure reduction, is still poorly understood [2]. Additional research projects include multiple system atrophy, and autonomic functions such as blood pressure management.

## VI Limitations of the Algorithmic Approach

An important technical constraint of the DyNeuMo system is that sensor measurements are restricted to the implantation site, limiting peripheral feedback when the device is cranially mounted for DBS therapy [14]. Another limitation is the priority handling of interrupts for balancing slow- and fast-acting algorithms. At this time, we simply prioritize the latest interrupt. Applications might warrant setting alternative priority approaches, or adding additional masks that combine the multiple classifier outputs to determine the final stimulation parameter set. Finally, our mapping of the algorithm source to a specific stimulation program limits the accessible therapy space. At this time, we favor this constraint to contain the risk of the adaptive algorithm.

Physiological factors might also limit the utility of our adaptive algorithms. The temporal dynamics between stimulation and physiological response need to align – if stimulation requires extended time to take effect, the utility of fast-adaptive titration could be diminished. The current device only supports a circadian or sub-circadian (ultradian) clock pattern; infradian rhythms such as those implicated in epilepsy require a pattern update from the clinician programmer. In the future, we can imagine using sensor measurements to fine-tune the “phase” of the time-based scheduler, much as light exposure serves to reset phase in healthy circadian rhythms. Longitudinal data will allow us to refine future designs based on actual real-world experience.

## VII Summary

The design of adaptive systems has the potential to impact the treatment of neurological disorders with complex dynamics by utilising sensor-based data and brain signals to adjust to patient-needs on multiple timescales. The additional consideration of circadian rhythms might also allow the stimulation state to take into account the dose-response-time of medication throughout the day, improving synergy between treatment modalities. Patients might also benefit from reduced side effects impacting sleep. To explore adaptive neuromodulation merging sensor- and time-based inputs, we have developed a fully-implantable research tool for investigational studies. While our initial clinical research focuses on diseases of the central nervous system, the DyNeuMo Mk-2 research tool should also prove useful for research in peripheral neuromodulation [31], as other organ systems might also benefit from a circadian component of therapy management.

## Figures and Tables

**Fig. 4:**
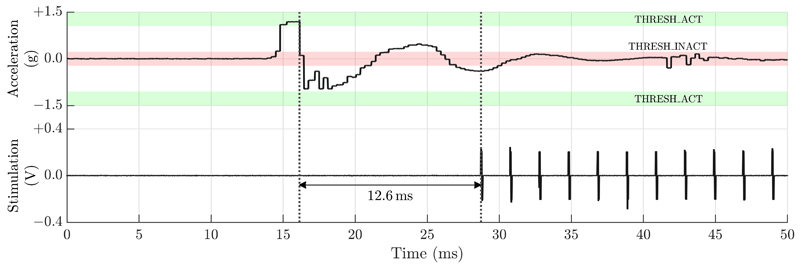
Initiation of stimulation due to a transient shock, that ceases after a programmed time of inactivity. The rapid response time of 12.6 ms helps support time-critical interventions. Figure reproduced from [14].

**Table I:** Definition of DyNeuMo Mk-2 algorithm system requirements; full requirements can be found in [14]

User Needs	
Predicate Therapy Support	The research system must support existing stimulation parameters for therapy delivery (amplitude, frequency, pulse width)
Slow-Adaptive Stimulation Scheme	Stimulation based on assignment of discrete stimulation parameters to specific time intervals in throughout the circadian cycle. Temporal mapping facilitated through the clinician programmer
Fast-Adaptive Sensing Scheme	Inertial accelerometer (three axis) – with DC accuracy for posture detection and AC capability for activity, tremor, gait, shocks and free-fall – flexibility for configuration to specific therapy needs; fully configurable through telemetry update
	Biopotential amplifier – local field potentials measured from implanted leads, including spectral power analysis or evoked potentials; fully configurable through telemetry update
Algorithm Methods and Priority	Slow-adaptive and Fast-adaptive algorithms classify a defined state and map this state to a specific stimulation parameter set pre-defined by the clinician. Priority is currently defined to the latest algorithm interrupt; upon termination of the fast-adaptive state, the signal will return to the slow-adaptive setting
Algorithm Power Allowance	Desired: the adaptive algorithm must draw no more than 25% of the nominal therapy power (e.g. 100μW for deep brain stimulation). Mandatory: the power consumption will not require in excess of a daily recharge
Algorithm Slow-Adaptive Granularity	Stimulation epochs will be provided in a 30 minute (max) intervals through a 24-hour calendar
Algorithm Fast-Adaptive Latency	< 20 ms from event detection to stimulation adjustment
Algorithm Risk Mitigations	Please reference [17] [14] for an overview of therapy limits, ramp rates, and fallback modes used for algorithms

**Table II:** Verification of the DyNeuMo Mk-2 algorithm requirements; additional results can be found in [14]

Sensor Characteristics	
Inertial sensing	3-axis accelerometer, sensitive to 4 mg activity variations; dynamic range programmable ±2 g to ±16 g; typical sampling rate is 50 Hz
Bioelectrical sensing	Multiplex bipolar connections across all electrodes (typically two leads, four contacts each) and to the case, estimated noise floor is approximately 1 μV rms signal in presence of 4mA stimulation; typical sampling rate is 625 Hz (multiple of stimulation frequency)
**Stimulation Characteristics**	
Stimulation Control policy	Detected classification states mapped to pre-configured stimulation programs with pre-specified transition ramp rate. Priorities and multi-detector program decisions based on clinician configuration table.
**Slow-Adaptive Algorithm**	
Circadian Scheduler	Twenty-four hour array with 30 minute default time epochs (firmware adjustable to 30 seconds); unique stimulation pointer provided for each epoch
**Fast-Adaptive Algorithm**	
Motion Classification	Orientation, activity/non-activity (parameterized), shocks and free-fall
Field Potential Classification	Configurable digital filtering (e.g. biquad and exponential filters, absolute value), time- or spectral-thresholds, and transition timing logic
